# Muscle quality determined by computed tomography predicts short-term and long-term survival after liver transplantation

**DOI:** 10.1038/s41598-023-33349-y

**Published:** 2023-05-10

**Authors:** Isabel Molwitz, Franziska Recklies, Maria Stark, Thomas Horvatits, Johannes Salamon, Samuel Huber, Lutz Fischer, Gerhard Adam, Ansgar W. Lohse, Martina Sterneck, Karoline Horvatits

**Affiliations:** 1 Department of Diagnostic and Interventional Radiology and Nuclear Medicine, University Medical Center Hamburg-Eppendorf, Martinistraße 52, 20246 Hamburg, Germany; 2 I. Department of Medicine, University Medical Center Hamburg-Eppendorf, Martinistraße 52, 20246 Hamburg, Germany; 3 Institute of Medical Biometry and Epidemiology, University Medical Center Hamburg-Eppendorf, Hamburg, Germany; 4 Department of Visceral Transplantation, University Medical Center Hamburg-Eppendorf, Hamburg, Germany

**Keywords:** Gastroenterology, Hepatology, Medical research, Outcomes research, Nutrition, Prognosis

## Abstract

Sarcopenia, the loss of muscle mass and quality, contributes to worse clinical outcome in patients with end-stage liver disease, but its impact on short- and long-term survival remains insufficiently understood. The aim of this study was to evaluate the development of computed tomography (CT) muscle parameters and their impact on short-term and long-term survival after liver transplantation. This retrospective study included patients with liver transplantation between 2011 and 2015 and a pre-transplant CT scan. Clinical characteristics, CT muscle mass and density were assessed pre-transplant, and in available CT scans at short-term (11 months) and long-term follow-up (56 months). Overall, 93/152 (61%) patients (109 male, 55 ± 10 years) suffered from sarcopenia pre-transplant. In short- (n = 50) and long-term follow-up (n = 52) the muscle mass (− 2.65 cm^2^/m^2^ 95% CI [− 4.52, − 0.77], p = 0.007; − 2.96 cm^2^/m^2^ [− 4.7, − 1.23], p = 0.001, respectively), and muscle density (− 3 HU [− 6, − 1], p = 0.007; − 2 HU [− 4, 0], p = 0.069) decreased. Myosteatosis was associated with a higher post-transplant mortality (survival probability: 3 months 72% vs. 95%, 1 year 63% vs. 90%, 5 years 54% vs. 84%, p = 0.001), while muscle mass was not. In conclusion, muscle mass and quality did not improve after transplant. Muscle quality predicts short- and long-term survival and could help to identify a patient’s risk profile.

## Introduction

Sarcopenia is a progressive skeletal muscle disorder, which is associated with adverse outcome, e.g., functional decline, frailty, and mortality. Aside from primary sarcopenia in aging, it can occur secondary, as a consequence of chronic diseases^1^. According to the revised consensus of the European Working Group on Sarcopenia in Older People 2 from 2019 it is recommended to confirm the diagnosis of suspected sarcopenia in patients with low muscle strength by measuring muscle quantity and quality^1^. In patients awaiting liver transplantation (LT) low muscle mass is prevalent in up to 70%^2,3^. Decreased muscle mass has been described as an independent factor for waiting list and posttransplant mortality as well as for longer intensive care unit (ICU) and hospital stay after transplantation^2,4–6^. Likewise, myosteatosis, which is an indicator of low muscle quality^7^, has been found to be associated with adverse outcome in patients with LT, such as higher perioperative mortality and longer hospital stay^8–10^. Moreover, in patients awaiting LT low muscle mass and quality were described as independent risk factors for hepatic encephalopathy^11^. Because of the relevance of sarcopenia in patients with chronic liver disease, the European Association for the Study of Liver Diseases implemented the assessment of sarcopenia and strategies to improve muscle mass and function into their guidelines in 2019^12^.

Muscle strength as a first indicator of sarcopenia can be assessed easily by handgrip strength, and physical performance, which determines severity of sarcopenia, by, e.g., gait speed^1^. To measure muscle mass and quality there are a variety, however, somewhat limited techniques: in bioelectrical impedance analysis results are biased by a patient’s hydrational status and time of day^13^, dual-energy absorptiometry as a two-dimensional technique relies on assumptions of the body compartments, while ultrasound delivers at best semi-quantitative results^14^. Magnetic resonance imaging and computed tomography (CT) are thus considered the reference standard^1^. While magnetic resonance imaging is a resource-intense, potentially burdensome examination for seriously ill patients, it is beneficial that most patients with end-stage liver disease already receive CT scans during the transplant evaluation, e.g., to exclude a hepatocellular carcinoma (HCC). In CT scans the muscle mass can easily be determined by the skeletal muscle area (SMA). Myosteatosis as a measure of muscle quality can be indirectly assessed by the muscle radiodensity attenuation^1^ (MRA) or quantified directly by material decomposition in spectral CT techniques^15,16^.

While the prognostic relevance of sarcopenia as a common comorbidity of liver cirrhosis is well known^6,17–21^, the literature on regeneration of muscle mass and quality after LT remains contradictory^22–24^. Only few studies, which focused on the first 1–2 years after liver transplantation, have assessed the course of CT muscle mass and density pre- and post-LT^19–24^. Reported results on long-term assessment are based on non-radiological methods such as bioelectrical impedance analysis^25,26^. Moreover, concerning the impact of pre-LT sarcopenia on post-LT survival, most studies investigated the CT muscle mass^2^, but did not include the muscle density, or the employed measurement techniques to determine CT muscle mass and quality varied from the recommended approach^27,28^.

Therefore, the aim of this study was to evaluate the course of both CT muscle mass and density as objective parameters of muscle quantity and quality after LT and their impact on short-term and long-term survival post-LT.

## Methods

### Study design and clinical data collection

This retrospective observational study was approved by the local ethics committee of the Ärztekammer Hamburg, Germany with available informed written consent of the participants or their legal guardian (No. WF-191/20) and conducted in compliance with the latest Declaration of Helsinki. The manuscript is reported in accordance with the STROBE guidelines.

**Figure 1:**
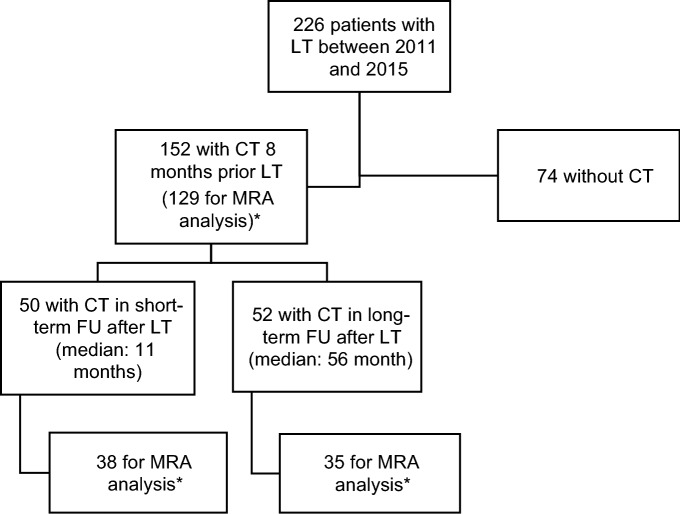
Flowchart of patient inclusion. *LT* liver transplantation, *CT* computed tomography, *MRA* muscle radiodensity attenuation, *FU* follow-up. *For analyses of the muscle radiodensity attenuation (MRA) only CT scans in a venous scan phase were employed to avoid a bias due to contrast agent dependent changes of tissue density^31^ (for further details please refer to the method section).

Inclusion criteria were: (a) liver transplantation between 2011 and 2015, (b) age ≥ 18 years, and (c) a CT scan within 8 months prior to LT. Exclusion criteria were: (a) CT scans with major artifacts or (b) CT scans with incomplete display of the abdominal muscle wall. A flow chart of patient inclusion is displayed in Fig. 1.

Short-term follow-up (FU) was defined as 6–18 months post-LT, and long-term FU as ≥ 19 months post-LT. Patients who died during post-LT ICU stay were censored after death.

For all included patients, demographic data, the type of liver disease, the pre-LT model of end-stage liver disease (MELD) score, a decompensation of liver disease, the body mass index (BMI), preexisting medical conditions, the pre-LT Charlson Comorbidity Index (CCI), and pre-LT renal parameters were collected. Patients with a BMI < 18.5 kg/m^2^ were classified as underweight according to the World Health Organization’s definition^29^. For patients on renal replacement therapy, which biases renal blood parameters, serum creatinine was normalized at 5.1 mg/dl and the glomerular filtration rate (GFR) at 9.9 ml/min. Post-LT, grading of post-operative complications according to the Clavien-Dindo classification within the first 90 days after transplant, length of ICU stay, time to death, as well as, the BMI, and CCI at the time of each CT scan were noted. Additionally, data about graft failure was collected.

### CT muscle mass and muscle quality assessment

Axial CT slices at the level of the 3rd lumbar vertebrae (L3) were extracted from the clinical picture archiving and communication systems (PACS) and further processed with the open-source software ImageJ (National Institutes of Health and the Laboratory for Optical and Computational Instrumentation, Wisconsin, USA)^30^. CT scans (iCT, Philips, Best, the Netherlands) were performed with 120 kV. Reconstructed slice thickness was 5 mm.

**Figure 2:**
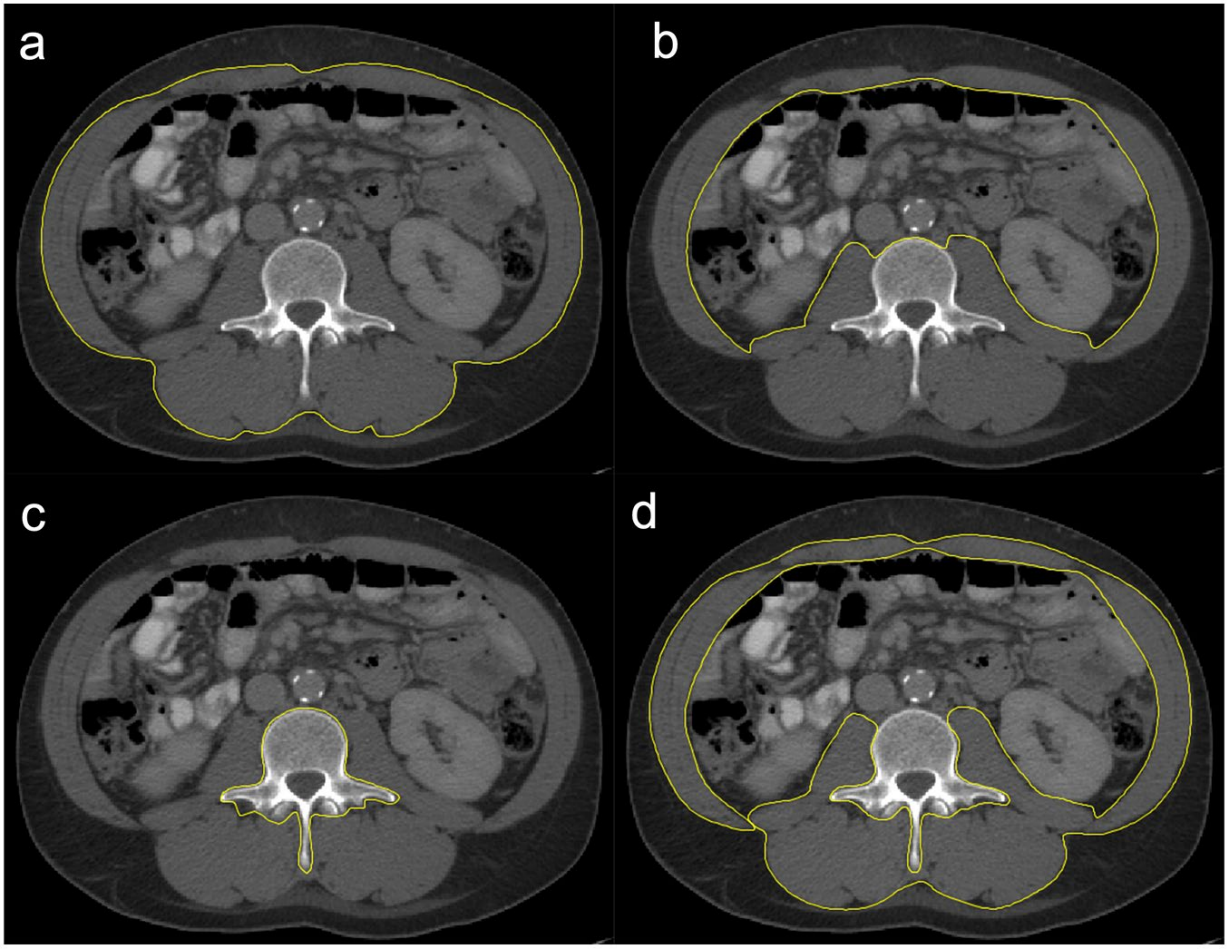
Regions of interest for CT muscle mass and muscle density assessment. Exemplary definition of regions of interest (ROIs) in a patient with high muscle status. ROIs were drawn (**a**) along the outer circumference of the whole abdominal muscle at the level of the third lumbar vertebral body (L3), (**b**) along the inner circumference of the abdominal muscle and L3, and (**c**) around L3. The skeletal muscle index (SMI) was calculated by: area of (ROI 1– ROI 2– ROI 3 [cm^2^])/(body height [m])^2^ after application of a muscle specific threshold of − 29 to + 150 Hounsfield units (HU). The muscle radiodensity attenuation in HU-values was derived from a ROI around the whole abdominal muscle area (**d**) after application of the muscle specific threshold.

For the assessment of the skeletal muscle mass all available CT scans were used. For muscle mass assessment the scan phase is irrelevant^31^. Thus, if the employed CT scans were multiphasic, the venous scan phase was preferably used to be coherent to the muscle density analyses (see below). Otherwise, other available CT phases were employed. To determine the skeletal muscle mass regions of interest (ROI) were defined along the outer parameter of the whole abdominal muscle (ROI 1), the inner circumference of the abdominal muscle including L3 (ROI 2), and along the outer parameter of L3 (ROI 3) (Fig. 2a–c). The SMA was then calculated by subtracting the area of ROI 2 and 3 from ROI 1 after setting a muscle-specific threshold of − 29 to + 150 Hounsfield units (HU) according to Gomez-Perez et al.^32^. From the SMA the skeletal muscle index (SMI) was derived by correction for square body height (SMA/body height^2^). Because cut-off values to determine sarcopenia based on the SMI differ between ethnics and depend on the entity, the SMI cut-off values of Carey et al. (male < 50 cm^2^/m^2^, female < 39 cm^2^/m^2^) which were specifically defined in a collective of LT-patients were applied^33^.

To measure muscle quality the MRA in HU-values was determined. Contrary to the skeletal muscle mass, the MRA changes between non-contrast-enhanced and contrast-enhanced scans^31^. As in this study’s cohort, the venous scan phase was most frequently retrospectively available, MRA analyses were thus exclusively performed on venous CT scans. All other CT examinations were excluded for MRA analyses. For MRA analyses another ROI was defined around the whole abdominal muscle circumference (Fig. 2d). The MRA was derived by noting HU values within this ROI after application of the muscle-specific threshold to exclude voxels with extramyocellular fat. Proposed MRA cut-off values to detect patients with sarcopenia in other entities mostly depend on the BMI^34,35^. However, because of hydropic decompensations with edema and ascites which influences the BMI in liver transplant candidates, the use of such cut-off values may be biased. Therefore, patients were assigned to a group below and above the mean MRA within this study’s collective. Because metric analyses of muscle parameters potentially provide a better understanding of their impact than cut-off-based approaches^36^ continuous analyses of SMI and MRA data were performed, as well.

### Statistical analyses

Categorical variables are presented with absolute and relative frequencies, continuous variables with mean and standard deviation if normally distributed or median and interquartile range (IQR) for skewed data distribution. Data distribution (normal vs. skewed) was tested by histograms.

To analyze changes of the SMI and MRA from pre-LT to short-term FU and pre-LT to long-term FU, a univariate linear regression model was used, respectively. The dependent variable was the difference between the pre-LT and respective post-LT values. The pre-LT value was included for baseline adjustment. No analyses were performed to compare the change of the SMI and MRA between short-term FU and long-term FU as not all patients with a CT scan during the long-term FU interval also received a CT scan in the short-term FU. Because ascites and edema may influence the SMA and thus muscle mass assessment, additionally, potential differences in the development of muscle mass pre-LT to short-term FU and pre-LT to long-term FU between the group of patients with and the group of patients without ascites or edema were evaluated using a t-test.

For group comparison, of e.g., patients below and above the SMI cut-off or patients with and without dialysis, the chi-square test, t-test, and Pearson correlation for normally distributed variables were employed. For skewed data distribution the Mann–Whitney-U-test and Spearman’s correlation were employed.

For analyses on graft survival, Kaplan–Meier estimators between patients below and above the SMI cut-off and below and above the mean MRA of the study collective were calculated and compared using the log rank test. Deceased patients at each time point (3 months, 1 year, 5 years post-LT) were censored. For analyses concerning length of ICU stay and survival, multivariable Cox proportional hazards regressions were used, with sex, age, MELD score, BMI, as well as metric and categorized SMI and MRA as potential predictors and adjusting parameters. Additionally, for analyses on length of ICU stay dichotomization results of patients based on their postoperative complications according to the Clavien–Dindo classification within 90 days post-LT (< and ≥ grade IIIb) were integrated as adjusting parameter. Grade IIIb was chosen as cut-off, as this indicates necessary intervention under general anesthesia and thus severe complications which may increase length of ICU stay^37^. As a Clavien-Dindo score ≥ IIIb already includes death this was no adjusting parameter in survival analyses. Additionally, the Kaplan–Meier estimators were determined between patients below and above the SMI cut-off and the group below and above the mean MRA. Differences between the groups were verified by the log-rank test.

For some patients at short-term FU and long-term FU no information about the MRA was available, because the MRA was only determined on CT scans within the same contrast phase to avoid a bias of muscle density caused by contrast agent^31^. All MRA analyses are thus based on the number of patients with available CT scans for MRA analyses (Fig. 1).

As SMI cut-off values differ between men and women, all analyses were additionally conducted for the subgroup of male patients. For male subgroup analyses concerning the MRA, the pre-LT mean MRA of the male patient collective was employed (38 HU), which was identical to the mean MRA of the whole study collective (38 HU). No separate analyses were conducted for the subgroup of female patients due to insufficient group size. For statistics IBM SPSS, Version 27 (SPSS Inc., Chicago, IL, USA) was used. All p values are considered descriptive. Statistical analyses were planed and verified by the statistician M.S.

## Results

### Study collective

Of the 152 included patients (109/152 (72%) male, mean age 55 ± 10 years) mean BMI pre-LT was 25.3 ± 5.4 kg/m^2^ and median MELD score 20 (IQR 12–30). Most patients (53/152, 35%) suffered from alcoholic liver disease, followed by viral hepatitis (34/152, 22%), and autoimmune liver diseases (19/152, 13%). Concomitant HCC was prevalent in 49/152 (32%) patients pre-transplant, in 29/50 (58%) patients with short-term FU and 28/52 (54%) patients with long-term FU post-LT. The distribution of all variables pre-LT, at short-term FU, and long-term FU is listed in Table 1.

**Table 1:** Patient characteristics prior to liver transplantation, in the group of patients with CT scans in the short-term and long-term follow-up after transplantation.

Patient characteristic	Pre-LT			Short-term FU post-LT			Long-term FU post-LT		
Sex	Total (n = 152)	Male (n = 109)	Female (n = 43)	Total (n = 50)	Male (n = 37)	Female (n = 13)	**Total (n = 52)**	**Male (n = 36)**	**Female (n = 16)**
Age (years)	55 ± 10	55 ± 10	55 ± 10	57 ± 8	57 ± 8	58 ± 10	60 ± 8	60 ± 8	61 ± 9
BMI (kg/m^2^)	25.3 ± 5.4	25.4 ± 5.2	25.0 ± 5.9	24.6 ± 4.2	25.3 ± 4	22.6 ± 4.2	25.0 ± 4	25.0 ± 3.8	25.5 ± 4.7
Underweight (BMI < 18.5 kg/m^2^)	7 (5)	3 (3)	4 (9)	3 (6)	–	3 (23)	-	-	-
MELD-score pre-LT	20 (12 – 30)	20 (13 –30)	24 (11 –32)	13 (9 – 26)	13 (9 – 23)	16 (9 – 28)	14 (9 – 28)	13 (8 – 24)	24 (11 –33)
CCI	0.0 (0 – 1)	0.0 (0 – 1)	0.0 (0 – 1)	1.0 (0 – 3)	1.0 (0 – 3)	1.0 (0 – 3)	2.0 (1 – 4)	2.5 (1 – 4)	2.0 (0 – 4)
Etiology of liver disease									
Alcoholic	53 (35)	38 (35)	15 (35)	20 (40)	15 (41)	5 (38)	23 (44)	17 (47)	6 (38)
Viral	34 (22)	24 (23)	10 (23)	18 (36)	14 (38)	4 (31)	17 (33)	13 (36)	4 (25)
Autoimmune	19 (13)	11 (10)	8 (19)	4 (8)	2 (5)	2 (15)	2 (4)	-	2 (13)
NASH	9 (6)	6 (6)	3 (7)	1 (2)	1 (3)	–	1 (2)	-	1 (6)
Re-transplantation	10 (7)	8 (7)	2 (5)	1 (2)	–	1 (8)	2 (4)	2 (6)	-
Acute liver failure	9 (6)	7 (6)	2 (5)	–	–	–	-	-	-
Cryptogenic or other	18 (12)	14 (13)	4 (9)	6 (12)	5 (14)	1 (8)	7 (14)	4 (11)	3 (19)
Concomitant HCC	49 (32)	37 (35)	12 (28)	29 (58)	22 (60)	7 (54)	28 (54)	23 (64)	5 (31)
Before LT									
Dialysis	32 (21)	17 (16)	15 (35)	5 (10)	3 (8)	2 (15)	7 (14)	1 (3)	6 (38)
Hepatorenal syndrome	47 (31)	28 (26)	19 (44)	11 (22)	5 (14)	6 (46)	10 (19)	4 (11)	6 (38)
Hepatic encephalopathy	78 (51)	58 (53)	20 (47)	21 (42)	17 (46)	4 (31)	23 (44)	15 (42)	8 (50)
Ascites	84 (55)	56 (52)	28 (65)	28 (56)	19 (51)	9 (69)	25 (48)	14 (39)	11 (69)
Esophageal varices	93 (61)	64 (59)	29 (67)	31 (62)	20 (54)	11 (85)	35 (67)	21 (58)	14 (88)

Data are displayed as absolute and relative frequencies, mean and standard deviation for normally distributed data and median with interquartile range for skewed data distribution.

*LT* liver transplantation, *FU* follow-up, *BMI* body mass index, *MELD* model of end-stage liver disease, *CCI* Charlson comorbidity index, *NASH* non-alcoholic steatohepatitis, *HCC* hepatocellular carcinoma.

### Pre-LT muscle mass and quality

Median time between the first CT scan and LT was 1 month (IQR 0–3). Mean muscle mass pre-LT was 44.59 ± 9.06 cm^2^/m^2^. According to the sex-specific muscle mass cut-off values 93/152 (61%) patients had sarcopenia pre-LT. These patients were less frequently suffering from non-alcoholic steatohepatitis (NASH) cirrhosis (n = 2) or acute liver failure (n = 2) than patients without sarcopenia (NASH: n = 7, acute liver failure: n = 7, difference between the group with and without sarcopenia: p = 0.013 for each entity). On the contrary, patients with pre-LT sarcopenia were more commonly suffering from alcoholic liver disease (n = 37) than patients without sarcopenia (n = 16, p = 0.110). There was no relevant association between patients with sarcopenia pre-LT and other etiologies of liver disease, age, MELD score, or concomitant HCC. The relation to all surveyed parameters, including pre-LT decompensation is displayed in Table 2.

**Table 2:** Patients characteristics before liver transplantation in the group of patients below and above the sarcopenia muscle mass cut-off value and below and above the mean muscle radiodensity attenuation (MRA) of this study’s population.

Patient characteristics	Non-sarcopenia^†^ (n = 59), n (%)	Sarcopenia^†^ (n = 93), n (%)	P value	MRA^‡^ ≥ 38 (n = 61), n (%)	MRA ‡ < 38 (n = 68), n (%)	P-Value
Age (years)	54 (11)	55 (9)	0.577	51 (11)	57 (8)	< 0.001
Male	38 (64)	71 (76)	0.111	49 (80)	45 (66)	0.071
BMI < 18.5 kg/m^2^	1 (2)	6 (7)	0.173	2 (3)	4 (5)	0.483
MELD-score	20 (10–30)	21 (13–31)	0.362	18 (12–30)	24 (16–33)	0.039
Etiology of liver disease						
Alcoholic	16 (27)	37 (40)	0.110	18 (30)	26 (38)	0.297
Viral	18 (31)	16 (17)	0.055	17 (28)	9 (13)	0.039
Autoimmune	5 (9)	14 (15)	0.232	11 (18)	6 (9)	0.123
Acute liver failure	7 (12)	2 (2)	0.013	3 (5)	5 (7)	0.567
NASH	7 (12)	2 (2)	0.013	1 (2)	7 (10)	0.042
Re-transplantation	3 (5)	7 (8)	0.554	4 (7)	6 (9)	0.631
Others	3 (5)	15 (16)	0.040	7 (12)	9 (13)	0.762
Concomitant HCC	24 (41)	25 (27)	0.076	24 (39)	10 (15)	0.002
Decompensation pre-LT						
Hepatic Encephalopathy	28 (48)	50 (54)	0.448	27 (44)	39 (57)	0.139
Ascites	28 (48)	56 (60)	0.123	26 (43)	44 (65)	0.012
Variceal bleeding	10 (17)	13 (14)	0.618	8 (13)	13 (19)	0.357
Hepatorenal syndrome	13 (22)	34 (37)	0.059	13 (21)	29 (43)	0.01
Serum creatinine (mg/dL)	1.3 (0.9–2.4)	1.5 (1.0–3.0)	0.228	1.3 (1.0–2.2)	1.6 (1.1–5.1)	0.047
GFR (ml/min)	60.5 (28.2–80.7)	49.5 (19.5–71.4)	0.242	59.6 (33.6–81.6)	42.3 (9.9–67.7)	0.019
Dialysis pre-LT	11 (19)	21 (23)	0.562	8 (21)	20 (29)	0.025
CCI pre-LT	0.0 (0–1)	0.0 (0–1)	0.871	0.0 (0–1)	1.0 (0–1)	0.025
Post-LT ICU-stay (days)	6 (3–15)	8 (4–20)	0.217	6 (3–12)	11.5 (5–23.5)	0.010
90d Clavien Dindo ≥ IIIb	31 (53)	54 (58)	0.504	29 (48)	48 (71)	0.008

For categorical data absolute and relative frequencies are provided, for normally distributed metric data mean with standard deviation and median with interquartile range for skewed data distribution. Chi-square tests, t-tests, and Mann–Whitney-U-tests were used for group comparison.

*BMI* body mass index, *MELD* model of end-stage liver disease, *NASH* non-alcoholic steatohepatitis, *HCC* hepatocellular carcinoma, *LT* liver transplantation, *GFR* glomerular filtration rate, *CCI* Charlson comorbidity index, *ICU* intensive care unit, *d* days.

^†^Sarcopenia cut off values by Carey et al.: skeletal muscle index (SMI) women < 39 cm^2^/m^2^, SMI men < 50 cm^2^/m^232^.

^‡^Mean of the MRA in 129 patients with available pre-LT scans in venous scan phase.

The mean muscle density pre-LT was 38 ± 8 HU. Patients with a muscle density below the mean (68/129, 53%) were older (57 vs. 51 years, p < 0.001) and had a higher MELD score (24 vs. 18, p = 0.039). Also, muscle density below the mean was more frequently found in patients with NASH cirrhosis (n = 7 vs. n = 1, p = 0.042), hepatorenal syndrome (n = 29 vs. n = 13, p = 0.01), and ascites (n = 44 vs. n = 26, p = 0.012). Patients with a muscle density below the mean suffered less frequently from viral liver disease (n = 9 vs. n = 17, p = 0.039), or concomitant HCC (n = 10 vs. n = 24, p = 0.002). Patients with a muscle density below the mean were more likely to suffer from severe post-operative complications with a Clavien–Dindo classification ≥ IIIb in the 90-days period after LT than patients with a muscle density above the mean (p = 0.008). Details on clinical parameters for patients below and above the SMI cut-off and below and above the mean MRA are listed in Table 2. Corresponding information for the male subgroup can be found in the supplements, Table 1.

### Development of muscle mass and quality in short-term follow-up

At the defined short-term FU interval 50 patients had an eligible CT scan to measure muscle mass (median 11 months post-LT [IQR 9–13]). Of these 38 CT scans included a venous scan phase and were employed to assess muscle density.

**Figure 3:**
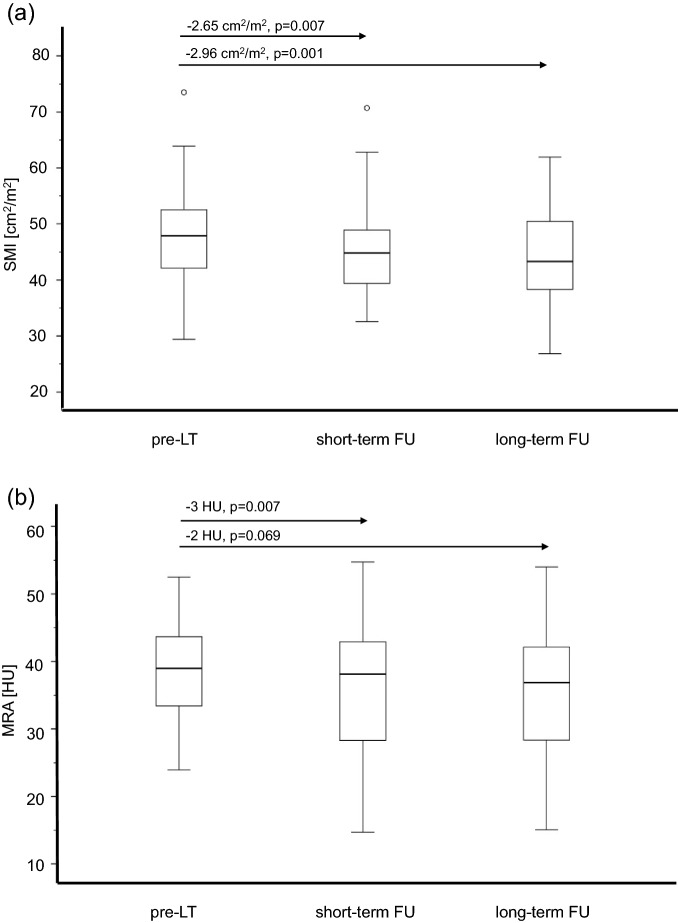
Development of the median skeletal muscle index (SMI) (**a**) and muscle radiodensity attenuation (MRA) (**b**) in short-term and long-term follow-up (FU) with mean differences and p values from the linear regression model. *LT* liver transplantation, *HU* Hounsfield units.

Mean muscle mass was 43.82 ± 8.61 cm^2^/m^2^, with thus a decrease of − 2.65 cm^2^/m^2^ (95% confidence interval (95% CI) [− 4.52, − 0.77], p = 0.007) compared to the pre-LT values within the same group of patients (Fig. 3a). Patients with ascites/edema (30/50, 60%) before LT showed a mean decrease of muscle mass of − 2.67 ± 8.22 cm^2^/m^2^. In patients without ascites/edema (20/50, 40%) pre-LT the muscle mass decreased by a mean of − 2.60 ± 7.87 cm^2^/m^2^. The difference in decrease between the group with and without ascites/edema was not relevant (p = 0.979). Based on the SMI cut-off values 35/50 (70%) patients were diagnosed with sarcopenia at short-term FU post-LT. Of those 14 patients had a newly developed sarcopenia, while four had recovered from sarcopenia since the pre-LT assessment.

Mean muscle density at short-term FU post-LT was 35 ± 10 HU. The muscle density thus decreased slightly by -3 HU (95% CI [− 6, − 1], p = 0.007) compared to pre-LT values in the same group of patients (Fig. 3b). At short-term FU 22/38 (58%) patients were below the defined pre-LT mean muscle density.

The development of muscle mass and density from pre-LT to short-term FU in the male subgroup showed a similar decrease as in the whole study cohort (Supplements, Table 2).

### Development of muscle mass and quality in long-term follow-up

At the defined long-term FU interval 52 patients had a suitable CT scan to assess muscle mass (median 56 months post-LT [IQR 40–74 months]). Of these 35 CT scans included a venous scan phase and were used to measure muscle density.

Mean muscle mass at long-term FU was 42.53 ± 8.46 cm^2^/m^2^ with thus a decrease of − 2.96 cm^2^/m^2^ (95% CI [− 4.7, − 1.23], p = 0.001) compared to pre-LT values in this patient group (n = 52) (Fig. 3a). Patients with ascites/edema (27/52, 52%) before LT showed a mean decrease of muscle mass of − 1.91 ± 7.35 cm^2^/m^2^ while muscle mass in patients without ascites/edema pre-LT (25/52, 48%) decreased by − 4.10 ± 7.19 cm^2^/m^2^. The difference in decrease between the group with and without ascites/edema was not relevant (p = 0.286). Based on the muscle mass cut-off at long-term FU 35/52 (67%) patients were classified with sarcopenia, of which eight patients had newly developed sarcopenia post-LT. Four had recovered from sarcopenia since the pre-LT muscle assessment.

Mean muscle density at long-term FU was 35 ± 9 HU, which represented no significant change compared to pre-LT muscle density values in the same group of patients (mean difference − 2 HU 95% CI [− 4, 0], p = 0.069) (Fig. 3b). At long-term FU 23/35 (66%) patients were below the defined pre-LT mean MRA value.

The development of muscle mass and density from pre-LT to long-term FU in the male subgroup showed a similar decrease as in the whole study cohort (Supplements, Table 2).

### Association of muscle mass and quality with clinical parameters

#### Muscle parameters and comorbidities

There was no correlation between the pre-LT MELD score and muscle mass or muscle density in the overall study collective. For the male subgroup a negative correlation between the pre-LT MELD score and the SMI in both short-term FU (r = − 0.530) and long-term FU (r = − 0.515) was observed. Neither pre-LT nor at short-term FU or long-term FU there was a correlation between the CCI and the CT muscle parameters in the overall or male subgroup analyses. Patients with hepatorenal syndrome pre-LT had a lower metric pre-LT muscle mass and muscle density than those without this complication (p = 0.023, p = 0.009). Furthermore, patients requiring dialysis prior to LT showed worse metric pre-LT muscle mass and muscle density (p = 0.028, p = 0.005). These associations were not found in the male subgroup analysis. For patients classified with sarcopenia by the pre-LT muscle mass cut-off no significant association was found to a hepatorenal syndrome, a lower GFR, or the necessity of dialysis (p = 0.059, p = 0.242, p = 0.562). Patients with a pre-LT muscle density below the mean had a lower GFR (p = 0.019) and were more frequently in need of dialysis (p = 0.025) (Table 2). Again, neither association was observed in the male subgroup analysis.

#### Muscle parameters and length of ICU stay

A Cox proportional hazards model including the categorical muscle mass below the SMI cut-off and muscle density below the mean with inclusion of adjusting variables (sex, age, MELD, BMI, Clavien–Dindo classification ≥ IIIb), revealed that a pre-LT muscle mass below the cut-off or pre-LT muscle density below the mean were not associated with an increase of the ICU stay (hazard ratio (HR) of ICU discharge 0.935 95% CI [0.596, 1.468], p = 0.770; HR 0.712 95% CI [0.436, 1.162], p = 0.174, respectively) (Table 3). Both a Clavien–Dindo classification ≥ IIIb and a higher pre-LT MELD-score were associated with prolonged ICU stay (HR of ICU discharge 0.203 95% CI [0.123, 0.334], p < 0.001; HR 0.976 95% CI [0.955, 0.998], p = 0.034, respectively).

**Table 3:** Cox proportional hazard model for length of stay at the intensive care unit.

	HR of ICU discharge [95% CI]	p value
Model with metric muscle parameters and BMI		
Age	0.970 [0.948–0.992]	0.007
Male sex	1.029 [0.640–1.654]	0.906
MELD-score	0.975 [0.953–0.997]	0.028
90d Clavien-Dindo ≥ IIIb	0.188 [0.114–0.311]	0.000
CCI	0.948 [0.766–1.174]	0.623
BMI	1.032 [0.981–1.085]	0.224
Metric MRA	1.006 [0.974–1.038]	0.730
Metric SMI	1.001 [0.972–1.032]	0.927
Model with categorial muscle parameters and underweight		
Age	0.978 [0.956–1.000]	0.052
Male sex	1.134 [0.706–1.821]	0.604
MELD-score	0.976 [0.955–0.998]	0.034
90d Clavien-Dindo ≥ IIIb	0.203 [0.123–0.334]	0.000
CCI	0.978 [0.787–1.216]	0.841
BMI < 18.5 kg/m^2^	0.471 [0.142–1.556]	0.217
MRA below the mean^‡^	0.712 [0.436–1.162]	0.174
SMI below the cut-off^†^	0.935 [0.596–1.468]	0.770

*HR* hazard ratio, *95% CI* 95% confidence interval, *MELD* model of end-stage liver disease, *d* days, *CCI* Charlson comorbidity index, *BMI* body mass index, *MRA *muscle radiodensity attenuation, *SMI* skeletal muscle index.

^‡^Mean of the MRA in 129 patients with available pre-LT scans in venous scan phase.

^†^Sarcopenia cut off values by Carey et al.: SMI women < 39 cm^2^/m^2^, SMI men < 50 cm^2^/m^2^^33^.

Also, in the Cox regression model with metric muscle parameters and adjustment for sex, age, MELD score, BMI, and Clavien–Dindo classification ≥ IIIb there was no association between the pre-LT muscle mass or pre-LT muscle density and length of ICU stay (HR of ICU discharge 1.001 95% CI [0.972, 1.032], p = 0.927; HR 1.006 95% CI [0.974, 1.038], p = 0.730, respectively) (Table 3). Again, in this model, a Clavien–Dindo classification ≥ IIIb was a predictor of length of ICU stay (HR of ICU discharge 0.188 95% CI [0.114, 0.311], p < 0.001) as were the pre-LT MELD score and age (HR of ICU discharge 0.975 95% CI [0.953, 0.997], p = 0.028; HR 0.970 95% CI [0.948, 0.992], p = 0.007, respectively). In the male subgroup analysis only the Clavien–Dindo classification ≥ IIIb was found to be a predictor for longer ICU stay in the model with the cut-off-based SMI/mean MRA (HR of ICU discharge 0.242 [0.135, 0.436], p < 0.001), as well as in the model with metric muscle values (HR of ICU discharge 0.199 [0.108, 0.364], p < 0.001).

#### Muscle parameters and graft survival

Post-LT 3-month graft survival rate was 90% (137/152), 1-year rate 90% (136/152), and 5-year rate 89% (135/172). There was no association of lower muscle mass (p = 0.504, p = 0.334, p = 0.208) or density (p = 0.211, p = 0.351, p = 0.244) with graft loss in the Kaplan Meier method with log rank test. In the subgroup analysis for male patients only a muscle density below the mean was associated with graft loss within 90 days post-LT (p = 0.030).

### Survival analysis

Among all 152 patients 3-month post-LT survival rate was 83%, 1-year survival 77% and 5-year survival 68%. Infection related complications were the most common cause of death (25/48, 52%), followed by graft or post-operative complications associated complications (6/48, 13%). The presence of sarcopenia (low muscle mass) pre-LT was not associated with an infectious cause of death (p = 0.153), while patients with a poor muscle quality (muscle density below the mean) died more frequently from infections than those with higher muscle density (p = 0.035).

In the multivariable Cox’ regression model a pre-LT muscle density below the mean increased the hazard of death (HR 2.985, 95% CI [1.365, 6.528]; p = 0.006) as did underweight (HR 3.185, 95% CI [1.023, 9.919]; p = 0.046). There was no association between a pre-LT muscle mass below the sarcopenia cut-off values and survival after LT (HR 0.979, 95% CI [0.481, 1.990]; p = 0.953) (Table 4).

**Table 4:** Cox proportional hazard model to describe the impact of clinical and CT muscle parameters on overall survival after liver transplantation.

	HR [95% CI]	p value
Model with metric muscle parameters and BMI		
Age	1.023 [0.982–1.066]	0.271
Male sex	0.706 [0.322–1.549]	0.385
MELD-score	1.026 [0.993–1.061]	0.125
CCI	1.151 [0.857–1.546]	0.351
BMI	0.990 [0.918–1.069]	0.806
Metric muscle density	0.945 [0.903–0.990]	0.016
Metric SMI	0.997 [0.953–1.043]	0.895
Model with categorial muscle parameters and underweight		
Age	1.021 [0.979–1.064]	0.336
Male sex	0.609 [0.287–1.292]	0.196
MELD-score	1.032 [0.998–1.066]	0.064
CCI	1.058 [0.785–1.425]	0.711
BMI < 18.5 kg/m^2^	3.185 [1.023–9.919]	0.046
Muscle density below the mean	2.985 [1.365–6.528]	0.006
SMI below cut-off	0.979 [0.481–1.990]	0.953

*HR* hazard ratio, *95% CI* 95% confidence interval, *MELD* model of end-stage liver disease, *CCI* Charlson comorbidity index, *BMI* body mass index, *SMI* skeletal muscle index.

**Figure 4:**
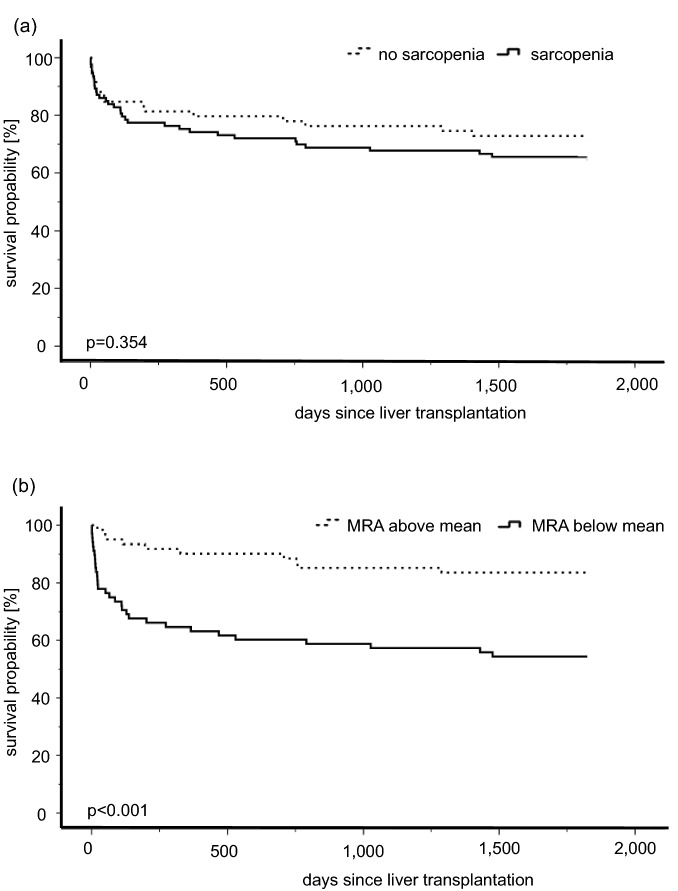
Overall survival of patients with and without sarcopenia according to the skeletal muscle index (SMI) cut-off of Carey et al. (**a**) or the muscle radiodensity attenuation (MRA) below and above the mean of the study collective (**b**) in the Kaplan Meier curve with log rank analysis.

The reduced survival rate of patients with a pre-LT muscle density below the mean was also displayed by the Kaplan–Meier curves (Fig. 4). Survival probability for patients with a pre-LT muscle density below the mean was 72% vs. 95% in the first 3 months after transplantation, 63% vs. 90% in the first year after LT, and 54% vs. 84% 5-years after LT (p < 0.001 respectively). The highest mortality rates for patients below the mean MRA were observed in the first 3 months after transplantation (Fig. 4b). Again, there was only a slight impact of sarcopenia according to the muscle mass cut-off on survival (Fig. 4a).

Concerning analyses with metric muscle parameters, the pre-LT muscle density slightly predicted overall mortality (HR 0.945, 95% CI [0.903, 0.990], p = 0.016), while the pre-LT muscle mass did not (Table 4). The survival analysis in the subgroup with male patients showed similar results (Supplements, Table 3 and 4).

## Discussion

This study analyzed the development of CT muscle mass and quality and their impact on short-term and long-term survival after liver transplantation.

The main findings were that (a) the skeletal muscle mass and muscle density, as a parameter of muscle quality, both decreased in the short-term FU post-LT as well as (b) from before transplantation to the long-term FU post-LT. With regards to survival (c) the CT muscle density as an indicator of muscle quality and myosteatosis had a relevant association with survival, especially within the first 3 months after LT, while (d) the CT muscle mass was not associated with short-term or long-term survival.

Concerning the development of body composition after LT body weight and adipose mass are known to increase^38,39^. However, literature on the development of lean muscle mass or myosteatosis after LT remains contradictory. Measured with varying techniques such as anthropomorphic methods, dual-energy absorptiometry, or CT inconsistent results have been published ranging from no relevant development^22^, to an increase of the prevalence of sarcopenia after transplantation^24^, to an improvement of muscle mass^23^.

Concerning studies which investigated changes from pre-LT CT muscle mass and density to post-LT results in short-term FU, Bhanji et al. described a median decrease of the SMI of 2.4 cm^2^/m^2^ (IQR − 0.9 to 5.6) as well as an increase in myosteatosis (− 5.0 HU [IQR − 8.6 to 0.1]) within 1 year post-LT in 161 patients^19^. This is in good agreement to this study’s results for short-term FU at a median of 11 months post-transplant (SMI: − 2.65 cm^2^/m^2^ [− 4.52, − 0.77]; muscle density: − 3 HU [− 6, − 1]). With a longer FU interval between 2.6 up to 29.3 months post-transplant Tsien et al. similarly described a decrease of the SMI from 25.9 ± 6.4 to 23.4 ± 5.2 cm^2^/m^2^ and a progress of myosteatosis with a decrease of muscle density from 15 ± 12 to 10 ± 13 HU in 53 patients^24^. Findings which are also supported by this study’s results for the SMI in long-term FU at a median of 56 months (IQR 40–74) post-LT (SMI: − 2.96 [− 4.7, − 1.23]). The muscle density in long-term FU in this study decreased as well, even if less pronounced, and not significantly (muscle density: − 2 HU [− 4, 0]). This may be because Tsien et al. used non-contrast enhanced CT scans, while measurements were conducted on venous CT scans in this study, in which the high density of contrast agent can cover small changes in the muscle density or may be due to the longer long-term FU interval in this study. While the best agreement to the whole-body muscle mass has been described for the whole abdominal muscle area at L3 as used in this study^27^, Jeon et al. measured sarcopenia by the area of the psoas muscle and similarly described an increase of patients with sarcopenia 12 months after LT^40^. Contradictory results have been published by Bergerson et al. who reported an increase of the SMI after LT^41^, in, however a small collective of 40 patients, with no assessment of myosteatosis, and no evaluation of the long-term course > 2 years after LT.

Therefore, in addition to existing studies on the course of CT muscle mass and CT muscle density in the short-term FU after LT, this study’s results demonstrate that the muscle mass and density remain impaired and even slightly decrease not only in short-term FU but also in the long-term FU post-LT.

With regards to the impact of pre-LT sarcopenia on survival many studies have assessed the influence of the CT muscle mass. In a meta-analysis including 19 studies a pooled HR of 1.84 (95% CI [1.11, 3.05], p = 0.02) was described for the impact of CT muscle mass on survival^2^. It should be noted that the measurement approach of the included studies was heterogenous: eight determined the SMI by the SMA of the whole abdominal muscle in the region of L3, while nine used the psoas muscle area. However, it has been described that the psoas muscle may be prone to atrophy in patients with concomitant diseases of the spine^42^, and that measurements of the psoas muscle are less suitable to detect mortality in waiting list patients^43^. It is generally recommended to measure the whole abdominal muscle area as done in this study^32^. Concerning this study’s results for the SMI, it was unexpected, that neither when employing the cut-off of Carey et al. which was especially designed for patients with end-stage liver disease, nor when using the metric SMI results a relevant relationship to survival was observed. This may be due to hydropic decompensation with ascites, which was present in 84 patients, causing water retention within the muscle fascia and thus biased SMA results. In comparable collectives of patients with end-stage liver disease and water retention the muscle density which is less susceptible to water retention could thus be especially relevant.

Studies which investigated the association of myosteatosis on post-LT survival are less common and most of those focused on the psoas muscle^6,24,44,45^. Coherent to this study’s results it has been recently shown by Cziganzy et al. that myosteatosis measured by the CT muscle density of the whole abdominal muscle area was significantly associated with patient survival (p = 0.001)^8^. Interestingly in that study, just as in our study no association was found between the SMI and patient survival (p = 0.278)^8^. Low muscle quality as indicated by myosteatosis may thus have a relevant and compared to muscle mass possibly more pronounced impact on survival in patients with liver transplantation.

This paper has several important clinical implications. In patients with suspected sarcopenia prior to LT, according to the guidelines of the European Association for the Study of the Liver it is recommended to improve the muscle status with dietary optimization, increased activity, physical exercises, and the treatment of the underlying disease^12^. As demonstrated by this study’s results, adherence to these guidelines could improve not only the waiting list survival but the outcome as well, especially in the first months but also up to years after transplantation. Concerning the necessary assessment of sarcopenia pre-LT, when employing CT muscle measurements, based on this study’s results not only the muscle mass but the muscle density should be assessed, as well. Furthermore, this study provides detailed information on how to assess the CT muscle mass and CT muscle density most reliably and in coherence to current recommendations.

Prospectively, it would be beneficial to also employ spectral CT scans such as dual-source, or dual-layer detector spectral CT, as those provide virtual non-contrast-enhanced images and thus contrast agent independent assessment of the muscle density. Also, in contrast to the indirect assessment of myosteatosis by muscle density, in spectral CT scans the muscular fat content can be directly assessed^15,16,46^ which further improves reliability of radiological myosteatosis assessment.

A limitation of this study is the reduced number of patients with CT scans in the short-term FU (n = 50) and long-term FU interval (n = 52). However, to avoid a bias, at both time points the development of the CT muscle mass and muscle density was compared to the CT values within the same group of patients before transplantation and no comparison was conducted between the different patient groups at short-term and long-term FU. Furthermore, survival analyses were based on all muscle mass and density values pre-LT. Therefore, the external validity of these analyses for comparable patient collectives should be given. Finally, one should note that this study investigated the patients’ muscle mass and quality, but there was no information on the patients’ physical performance by which severity of sarcopenia can be assessed^1^.

In this retrospective observational study, neither CT muscle mass nor CT muscle quality, as parameters of sarcopenia and myosteatosis, improved in short-term or long-term FU after liver transplantation.

In contrast to the muscle mass, muscle quality was found to be a prognostic factor for survival with a special impact on the first months post-LT. Therefore, in case of suspected sarcopenia in waiting list patients with available CT scans, not only the CT muscle mass, but CT muscle quality should be investigated, as well, to evaluate the patient’s risk profile and initiate appropriate nutritional regimes and physical therapy.

## Supplementary Information

Supplementary Information.

## Data Availability

The data used to support the findings of this study are available from the corresponding author at franziska.recklies@gmail.com upon request.
