# Supplementary material for: Muscle quality determined by computed tomography predicts short-term and long-term survival after liver transplantation
Source: Sci Rep. 2023 May 10;13:7631. doi: 10.1038/s41598-023-33349-y (PMC10172199; doi:10.1038/s41598-023-33349-y)
Supplement: Supplementary file 1 — Supplementary Information. [file 41598_2023_33349_MOESM1_ESM.pdf]

Supplemental results: subgroup analysis with male patients

Title: Muscle Quality determined by Computed Tomography predicts short-term and long-term Survival after Liver Transplantation

Isabel Molwitz[1]†, Franziska Recklies[2]*†, Maria Stark[3], Thomas Horvatits[2], Johannes Salamon[1], Samuel Huber[2], Lutz Fischer[4], Gerhard Adam[1], Ansgar W. Lohse[2], Martina Sterneck[2]†, Karoline Horvatits[2]†

†These authors contributed equally to the study.

[1] Department of Diagnostic and Interventional Radiology and Nuclear Medicine, University Medical Center Hamburg-Eppendorf, Hamburg, Germany

[2] I. Department of Medicine, University Medical Center Hamburg-Eppendorf, Hamburg, Germany

[3] Institute of Medical Biometry and Epidemiology, University Medical Center Hamburg- Eppendorf, Hamburg, Germany

[4] Department of Visceral Transplantation, University Medical Center Hamburg-Eppendorf, Hamburg, Germany

Table 1 Characteristics of male patients before liver transplantation in the group below and above the sarcopenia muscle mass cut-off value and below and above the mean muscle radiodensity attenuation (MRA) of this study's collective male patients

For categorical data absolute and relative frequencies are provided, for normally distributed metric data mean with standard deviation and median with interquartile range for skewed data distribution. Chi-square tests, t-tests and Mann-Whitney-U-tests were used for group comparison.

**Patient characteristics**	**Non-sarcopenia † (n=38), n (%)**	**Sarcopenia † (n=71), n (%)**	**P-Value**	**MRA ‡ ≥ 38 (n=49), n (%)**	**MRA ‡ < 38 (n=45), n (%)**	**P-Value**
Age (years)	54 (10)	55 (10)	0.361	51 (11)	57 (8)	0.007
BMI < 18.5 kg/m^2	0 (0)	3 (4)	0.199	0 (0)	3 (7)	0.066
MELD-score	18.4 (9-28.5)	20 (14-30.2)	0.234	18 (12-30.2)	23 (15.5-31)	0.127
**Etiology of liver disease**						
Alcoholic	11 (29)	27 (38)	0.343	13 (27)	16 (36)	0.344
Viral	13 (34)	12 (17)	0.041	17 (25)	4 (9)	0.003
Autoimmune	1 (3)	10 (14)	0.059	7 (14)	4 (9)	0.416
Acute liver failure	5 (13)	2 (3)	0.036	2 (4)	4 (9)	0.341
NASH	4 (11)	2 (3)	0.093	1 (2)	5 (11)	0.072
Re-transplantation	2 (5)	6 (8)	0.543	3 (6)	5 (11)	0.387
Others	2 (5)	12 (17)	0.084	6 (12)	7 (16)	0.642
Concomitant HCC	19 (50)	19 (27)	0.015	20 (41)	7 (16)	0.007
**Decompensation pre-LT**						
Hepatic Encephalopathy	18 (47)	40 (56)	0.371	23 (47)	27 (60)	0.205
Ascites	16 (42)	41 (58)	0.119	20 (41)	28 (62)	0.038
Variceal bleeding	5 (13)	10 (14)	0.894	6 (12)	9 (20)	0.305
Hepatorenal syndrome	6 (16)	22 (31)	0.084	11 (22)	15 (33)	0.239
Serum creatinine (mg/dL)	1.3 (1-2.2)	1.5 (1-2.8)	0.424	1.3 (1-2.5)	1.6 (1.2-4)	0.133
GFR (ml/min)	61.3 (33.1-80.3)	50.6 (25.2-79.5)	0.369	59.6 (30.2-80.47)	46.2 (16.2-68.3)	0.117
Dialysis pre-LT	4 (11)	13 (18)	0.286	6 (12)	10 (22)	0.199

CCI pre-LT	0.5 (0-1)	0.0 (0-1)	0.756	0.0 (0-1)	1.0 (0-1)	0.013
Post-LT ICU-stay (days)	5 (3-9)	8 (4-24)	0.038	6 (4-11)	10 (4-26.5)	0.110
90d Clavien Dindo ≥ IIIb	42 (59)	19 (50)	0.359	24 (49)	31 (69)	0.05

Abbreviations: MRA, muscle radiodensity attenuation; BMI, body mass index; MELD, model of end-stage liver disease; NASH, non-alcoholic steatohepatitis; HCC, hepatocellular carcinoma; LT, liver transplantation; GFR, glomerular filtration rate; CCI, Charlson comorbidity index; ICU, intensive care unit; d, days.

† Sarcopenia cut off values by Carey et al: skeletal muscle index (SMI) men < 50 cm^2/m^2 [33]

‡ Mean of the MRA in 129 patients with available pre-LT scans in venous scan phase

Table 2 Development of muscle mass and quality in short-term and long-term follow-up (FU) compared to pre liver transplant (pre-LT) values, estimated with a linear regression model.

**Short-term FU**			
**Muscle mass (n=37)**			
Mean SMI at short-term FU	Mean difference of SMI at short-term FU to pre-LT SMI	95%-CI	P-Value
45.85 ± 8.7 cm^2/m^2	-3.09 cm^2/m^2	[-6.16; -1.28]	0.004
**Muscle quality (n=28)**			
Mean MRA at short-term FU	Mean difference of MRA at short-term FU to pre-LT MRA	95%-CI	P-Value
36 ±10 HU	-2 HU	[-7; -1]	0.023
**Long-term FU**			
**Muscle mass (n=36)**			
Mean SMI at long-term FU	Mean difference of SMI at long-term FU to pre-LT SMI	95%-CI	P-Value
43.71 ± 8.98 cm^2/m^2	-3.8 cm^2/m^2	[-6.54; -1.98]	0.001
**Muscle quality (n=23)**			
Mean MRA at long-term FU	Mean difference of MRA at long-term FU to pre-LT MRA	95%-CI	P-Value
36 ± 9 HU	-2 HU	[-5; 0.2]	0.073

Abbreviations: SMI, skeletal muscle index; 95%-CI, 95% confidence interval; MRA, muscle radiodensity attenuation; HU, Hounsfield units

Table 3 Cox proportional hazard model to describe the impact of clinical and CT muscle parameters on overall survival after liver transplantation for the subgroup of male patients

	HR [95% CI]	P-Value
**Model with metric muscle parameters and BMI**		
**Age**	1.008 [0.963 – 1.054]	0.742
**Male sex**	-	-
**MELD-score**	1.020 [0.983 – 1.059]	0.290
**CCI**	1.319 [0.940 – 1.851]	0.110
**BMI**	0.987 [0.901 – 1.081]	0.780
**Metric MRA**	0.948 [0.899 – 0.999]	0.044
**Metric SMI**	0.990 [0.944 – 1.039]	0.691
**Model with categorial muscle parameters and underweight**		
**Age**	1.003 [0.958 – 1.051]	0.885
**Male sex**	-	-
**MELD-score**	1.028 [0.990 – 1.066]	0.148
**CCI**	1.307 [0.932 – 1.834]	0.121
**BMI < 18.5 kg/m^2**	6.834 [1.833 – 25.485]	0.004
**MRA below the mean‡**	2.510 [1.048 – 6.013]	0.039
**SMI below cut-off†**	1.115 [0.479 – 2.597]	0.801

Abbreviations: HR, hazard ratio; 95%-CI, 95% confidence interval; MELD, model of end-stage liver disease; CCI, Charlson comorbidity index; BMI, body mass index; MRA, muscle radiodensity attenuation; SMI, skeletal muscle index.

‡ Mean of the MRA in 129 patients with available pre-LT scans in venous scan phase

† Sarcopenia cut off values by Carey et al: SMI women < 39 cm^2/m^2, SMI men < 50 cm^2/m^2 [33]

Table 4 Kaplan-Meier curve with log rank test for mortality: number of deceased patients and results

	SMI below cut-off deceased/total (%)	SMI above the mean deceased/total (%)	P-Value
**90 days post-LT**	14/71 (20)	5/38 (13)	0.416
**1 year post-LT**	20/71 (29)	7/38 (18)	0.281
**5 years post-LT**	25/71 (35)	9/38 (24)	0.233
	**MRA below the mean deceased/total (%)**	**MRA above the mean deceased/total (%)**	**P-Value**
**90 days post-LT**	13/45 (29)	3/49 (6)	0.003
**1 year post-LT**	18/45 (40)	6/49 (12)	0.001
**5 years post-LT**	22/45 (49)	9/49 (18)	0.001

Abbreviations: LT, liver transplantation; SMI, skeletal muscle index; MRA, muscle radiodensity attenuation
